# Recent investigations on the impact of levonorgestrel and 17β-ethinylestradiol on melanoma

**DOI:** 10.25122/jml-2025-0125

**Published:** 2025-08

**Authors:** Florina Borozan, Alexandra-Denisa Semenescu, Larisa Tomescu, Ioan Sas

**Affiliations:** 1 Faculty of Medicine, Victor Babeș University of Medicine and Pharmacy, Timisoara, Romania; 2 Faculty of Pharmacy, Victor Babeș University of Medicine and Pharmacy, Timisoara, Romania; 3 Research Center for Pharmaco-Toxicological Evaluations, Faculty of Pharmacy, Victor Babeș University of Medicine and Pharmacy, Timisoara, Romania

**Keywords:** melanoma, proliferation, oral contraceptives, levonorgestrel, 17α-ethinylestradiol

## Abstract

Skin cancer is one of the most aggressive types of neoplasms, with high morbidity and mortality. Numerous factors are associated with the development of melanoma, including exposure to UV radiation and the use of exogenous hormones. Some studies suggest that oral contraceptives can influence the development of skin cancer, but the evidence is insufficient and contradictory. Our study aimed to initially evaluate the safety profile of the combination of levonorgestrel and 17β-ethinylestradiol on healthy cell lines (HaCaT and JB6 Cl 41-5a) and to further determine the possible association with skin cancer by investigating the effect of the combination on the murine melanoma tumor line (B164A5). The data obtained showed that levonorgestrel-ethinylestradiol (LG-EE) did not have a toxic effect on healthy cells (HaCaT and JB6 Cl 41-5a) but exhibited a slight proliferative effect on murine skin tumor cells at increasing concentrations. LG-EE on healthy lines did not significantly decrease viability and did not induce a cytotoxic effect. On the B164A5 tumor line, the hormonal combination at a concentration of 5 µM slightly decreased viability and degraded the cell membrane, observing a decrease in confluence and number of cells, as well as the presence of apoptotic bodies, while at the highest dose tested of 15 µM, an opposite effect was recorded with a slight stimulation of murine tumor cells. The results suggest that LG-EE may influence the development of melanoma; however, the evidence is insufficient, and further studies are necessary.

## INTRODUCTION

The World Health Organization (WHO) classifies combined oral contraceptives (COs) as Group 1 carcinogens, due to their influence on the occurrence of breast, cervical, and liver cancer [1]. Since their introduction, millions of women have used estrogen–progestin contraceptives for prolonged periods and at various doses. Both progestogens and estrogens can influence cell proliferation and are therefore considered potential contributors to carcinogenesis in susceptible tissues [2].

Among the most widely used components of COs are 17α-ethinylestradiol (EE), a synthetic estrogen first synthesized in 1938, and levonorgestrel (LG), a synthetic progestin [3,4]. Various studies have shown that combined contraceptives may increase the risk of developing cancer. Several studies have shown that combined contraceptives may increase the risk of certain cancers; however, this risk decreases after discontinuation, with baseline levels of risk usually restored after 5–10 years. Conversely, long-term CO use has been associated with a reduced risk of endometrial, ovarian, and colorectal cancers [5-7].

The relationship between cancer risk and COs use is complicated by various factors, such as the administration of multiple hormonal preparations, different compositions of formulations, and different concentrations, as well as aspects related to age at first pregnancy, number of pregnancies, breastfeeding, and sexual history [8]. Beyond gynecological cancers, evidence also suggests a possible association between CO use and melanoma. It has been shown that COs produce hyperpigmentation as an adverse effect, and estrogens influence the multiplication of melanocytes [9]. Furthermore, specific estrogen-binding receptors have been identified in melanomas and benign nevi [10].

Melanoma is one of the most aggressive types of cancer, which can develop in the skin, mucous membranes, and central nervous system, where melanocytic cells are found [11]. Its incidence has increased significantly in recent years, with high mortality rates and peak occurrence between ages 20 and 60 [12-15]. Melanoma has a strong genetic component; environmental and hormonal factors, including ultraviolet (UV) radiation and endogenous or exogenous hormones, also contribute to its pathogenesis [12,15,16].

The skin can synthesize estrogens, demonstrating their significant role in skin physiology. Estrogens influence the activity of melanocytes, keratinocytes, and fibroblasts, promote wound healing, and increase skin thickness in response to higher estrogen levels [17,18]. Estrogens can impact melanocyte proliferation and increase melanin production in the skin. The combination of estrogen-progestins induces melanogenesis, leading to increased melanin and hyperpigmentation. These effects may be present in pregnant women as well as in women taking COs or hormone replacement therapy [19].

Given the limited evidence available, our study aimed to assess the safety profile of the levonorgestrel and 17α-ethinylestradiol combination on two healthy cell lines, human keratinocytes and murine epidermal cells, and to evaluate its potential cytotoxic or proliferative effects on a murine melanoma cell line, to explore a possible association between contraceptive use and skin cancer.

## MATERIAL AND METHODS

### Chemicals, devices, and cell culture requirements

Different reagents were utilized for the in vitro tests. LG, EE, Dulbecco’s Modified Eagle Medium (DMEM), and Eagle's Minimum Essential Medium (EMEM) were acquired from Sigma Aldrich, Steinheim, Germany. Trypsin-EDTA, phosphate-buffered saline (PBS), penicillin/streptomycin (Pen/Strep) mixture, and fetal bovine serum (FBS) were procured from PAN-Biotech GmbH (Aidenbach, Germany). The lactate dehydrogenase (LDH) assay kit, MTT [3-(4,5-dimethylthiazol-2-yl)-2,5-diphenyltetrazolium bromide] kit, and Hoechst 33342 dye were purchased from Thermo Fisher Scientific (Waltham, MA, USA).

The HaCaT cell line was purchased from CLS Cell Lines Service GmbH (Eppelheim, Germany). The JB6 Cl 41-5a cells were obtained from American Type Culture Collection (ATCC, Lomianki, Poland), and the B164A5 tumor cells were acquired from Sigma Aldrich (ECACC), as frozen items and maintained in liquid nitrogen until used in experiments.

HaCaT and B164A5 cells were grown in a comprehensive medium, DMEM enriched with 10% FBS and 1% Pen/Strep, while JB6 Cl 41-5a murine cells were cultivated in EMEM supplemented with 5% FBS and 1% antibiotic solution.

The instruments used included an Olympus IX73 inverted microscope (Olympus, Tokyo, Japan), a Cytation 1 automated microscope, and a Cytation 5 plate reader (BioTek Instruments Inc., Winooski, VT, USA).

### Treatment protocol

The combination of LG and EE was dissolved in dimethyl sulfoxide (DMSO) to prepare a stock solution. Working concentrations of 5 µM, 7.5 µM, and 15 µM were freshly prepared for testing.

### Cell viability analysis

The effect of the LG–EE combination on cell viability was assessed using the colorimetric MTT assay. First, the safety profile of the combination was evaluated in two healthy cell lines (HaCaT human keratinocytes and JB6 Cl 41-5a murine epidermal cells). The same assay was then applied to murine melanoma B164A5 cells to determine cytotoxic or proliferative effects.

The viability analysis was performed by performing several steps: 1) initially healthy and cancerous cells (HaCat, JB6 CCl 41-5a, and B164A5) were cultured in special 96-well plates; 2) at the appropriate confluence (80-85%) the compounds (5, 7.5, and 15 µM) were applied to the cells; 3) after 24 hours of stimulation, 10 µL of reagent 1 was applied to each well and incubated for three hours; 4) the last step was the addition of 100 µL of solubilization buffer (reagent 2) for 30 minutes at room temperature and without light. After carrying out the working protocol, the absorbance values were read at 570 nm using Cytation 5 from BioTek Instruments Inc. (Winooski, VT, USA).

### Cytotoxicity assay– evaluation of LDH leakage

To determine the cytotoxic effect of the hormonal combination, the leakage of lactate dehydrogenase – a cytosolic enzyme observed following cell wall destruction – was quantified. After 24 hours of stimulation of cells (5, 7.5, and 15 µM) grown in 96-well plates, 50 µL of medium was transferred from each well to another plate, over which 50 µL of reaction mixture was applied, for 30 minutes at room temperature and protected from light, after which another 50 µL of stopping solution was added. Finally, the absorbances were measured at 490 and 680 nm using Cytation 5 from BioTek Instruments Inc. (Winooski, VT, USA). The percentage of cytotoxicity was calculated by quantifying LDH activity using the following formula from the manufacturer's protocol:

### Evaluation of cell morphology

The cell morphology of the three cell lines (HaCat, JB6 CCl 41-5a, and B164A5) was analyzed after 24 hours of treatment with the LG-EE combination (5, 7.5, and 15 µM). To determine the changes in morphology, cells were pictured under bright field illumination using an Olympus IX73 inverted microscope and a Cytation 1 automated microscope.

### Determination of cell confluence and cell number

B164A5 cancer cells were photographed (4x) after 24 hours of stimulation with LG-EE samples using a Cytation 1 microscope to determine cell number and confluence. The images were investigated using Gen5 Microplate Data Collection and Analysis Software, Version 3.14.

### Nuclear morphology assay – Hoechst method

The impact induced by LG-EE at the nuclear level in murine melanoma B164A5 cells was established by the Hoechst 33342 method. The cell line was grown in 12-well plates and then stimulated with LG-EE samples (5 and 15 µM) for 24 hours. The next day, the medium was removed, and Hoechst solution 1:2000 in PBS was applied for 10 minutes at room temperature and protected from light. Finally, the wells were cleaned with PBS (2x), and the labeled nuclei were photographed. Images were taken with a Cytation 1 microscope and analyzed using the Gen5 Microplate Data Collection and Analysis Software.

### Statistical analysis

The statistical differences between treatment groups were established using a one-way ANOVA test and Dunnett's multiple comparisons post-test. The program GraphPad Prism 9.4.0 (GraphPad Software, San Diego, CA, USA) was used to express the results as mean ± standard deviation (SD). The results were marked with * to specify statistically significant differences: * *P* < 0.05; ** *P* < 0.01; *** *P* < 0.001; **** *P* < 0.0001.

## RESULTS

### Cell viability assay

**Figure 1:**
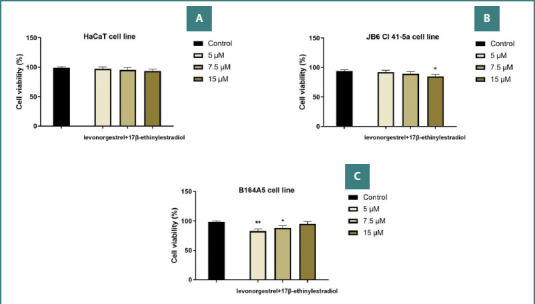
Cell viability of the LG–EE combination (5, 7.5, and 15 µM) assessed by the MTT assay 24 hours after treatment. A, HaCaT cells; B, JB6 Cl 41-5a cells; C, B164A5 cells. The statistical differences between the control and the stimulated group were examined by applying the one-way ANOVA test, followed by Dunnett’s multiple comparisons post-test (* P < 0.05; ** P < 0.01).

MTT analysis demonstrated that the LG–EE combination did not significantly reduce the viability of healthy cell lines. At the highest concentration tested (15 µM), viability remained at 93.61% for HaCaT cells and 84.92% for JB6 Cl 41-5a cells. Regarding the B164A5 melanoma line, a viability value of 82.55% was obtained at the 5 µM dose, followed by an increase with increasing dose, reaching 95.11% at 15 µM (Figure 1A-C), indicating slight proliferation.

### Cytotoxicity assay

**Figure 2:**
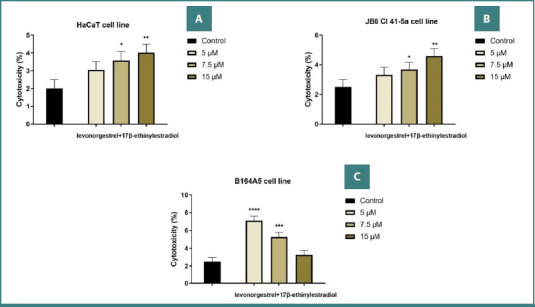
Cytotoxic effect of the LG–EE combination (5, 7.5, and 15 µM) 24 hours after treatment. A, HaCaT cells; B, JB6 Cl 41-5a cells; C, B164A5 cells. The statistical differences between the control and the stimulated group were examined by applying the one-way ANOVA test, followed by Dunnett’s multiple comparisons post-test (* P < 0.05; ** P < 0.01; *** P < 0.001; **** P < 0.0001).

LDH leakage was measured following treatment with the LG–EE combination to assess cytotoxicity. The results obtained are in accordance with previous data. Therefore, on keratinocytes and epidermal cells, a slight percentage increase was evident with increasing concentration, reaching percentages of ≈ 4.5% on both cell lines, with no cytotoxic effect present. On the other hand, on the mouse melanoma line, decreasing values were observed with increasing dose (up to 3.25%), suggesting that higher concentrations reduced the potential cytotoxic effect (Figure 2A-C).

### Cell morphology analysis

**Figure 3:**
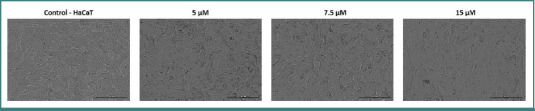
Morphological characteristics of HaCaT cells after 24 hours of treatment with the LG–EE combination (5, 7.5, and 15 µM). **Scale bars = 100 µm**.

**Figure 4:**
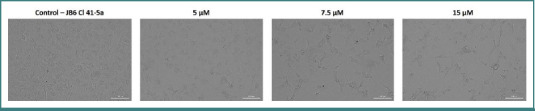
Morphological characteristics of JB6 Cl 41-5a cells after 24 hours of treatment with the LG–EE combination (5, 7.5, and 15 µM). **Scale bars = 100 µm**.

The next step was to analyze the impact of the LG-EE hormone combination on the morphology of the three cell lines. The initial analysis focused on HaCaT and JB6 Cl 41-5a cells (Figures 3 and 4). No adverse effects on cell shape were observed, although a slight, statistically insignificant reduction in cell confluence was noted.

**Figure 5:**
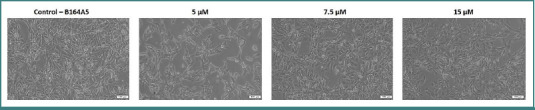
Morphological characteristics of B164A5 cells after 24 h of treatment with LG-EE combination (5, 7.5, and 15 µM). **Scale bars = 100 µm**.

Regarding the murine line B164A5 (Figure 5), a change in cell shape compared to the control was observed at the lowest concentration tested (5 µM), after which an increase in cell confluence was exhibited, with no alterations in cell shape being observed..

### Determination of confluence and cell number

**Figure 6:**
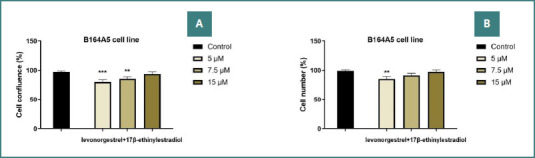
Confluence and cell number of B164A5 melanoma cells 24 hours after treatment with the LG–EE combination (5, 7.5, and 15 µM). A, Confluence percentage; B, Cell number. The statistical differences between the control and the stimulated group were assessed using one-way ANOVA with Dunnett’s post-test (**P < .01; **P < .001).

Following these data, the confluence and number of murine cells were additionally evaluated on the cancer line by applying the LG-EE combination (Figure 6 AB). With increasing LG–EE dose, both confluence and cell number rose (up to 97%), suggesting a slight proliferative effect on B164A5 cells at higher doses, whereas at lower doses, both parameters decreased.

### Nuclear staining assay

**Figure 7:**
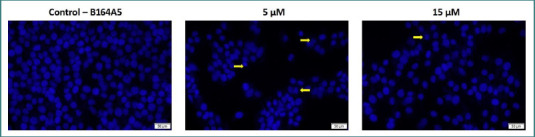
Nuclear morphology after 24 hours of treatment with the LG–EE combination (5 and 15 µM). **Yellow arrows indicate apoptotic features. Scale bars = 50 µm**.

Finally, the impact of LG-EE on the nuclear morphology of the B164A5 line cell was tested (Figure 7). The impact of the concentration of 5 µM and 15 µM, respectively, was evaluated. Changes in the structure of the nucleus shape were highlighted, more evident at the lowest concentration, with the presence of apoptotic bodies and nuclear condensation.

The analyses demonstrated that the LG–EE hormonal combination can induce dose-dependent in vitro proliferation of murine melanoma B164A5 cells.

## DISCUSSION

Although COs research largely focuses on reproductive effects, contextualizing findings within the broader field of oncology, such as melanoma, provides relevant clinical insights. Globally, melanoma had the highest incidence rates, with over 330,000 new cases in 2022 [20]. Diagnosis of the pathology is increasingly based on molecular criteria, going beyond morphology, to better guide targeted therapies [21]. Despite prevention campaigns, the global incidence of melanoma continued to rise, reflecting not only environmental risk factors but also earlier detection [22]. In recent years, significant advances have been made in melanoma treatment, including immune checkpoint inhibitors, targeted kinase inhibitors, and nanotechnology-based delivery systems [23].

Data from the literature suggest that COs may influence the development of melanoma. Still, there is no current data to show that either endogenous or exogenous hormones enhance the risk of skin cancer.

Estrogen use has been linked to an increased risk of developing melanoma. Nonetheless, melanomas with superficial spread often have a relatively favorable prognosis. Epidemiological data also suggest a bidirectional relationship between breast cancer and cutaneous melanoma, as women with breast cancer have a higher risk of subsequently developing melanoma, and vice versa [24-26].

Following these data, several cohort studies have evaluated the association between COs use and the evolution of melanoma. Early reports, published nearly 50 years ago, indicated that women using oral contraceptives had up to a fourfold higher risk of developing melanoma compared with non-users [27-30]. In contrast, more recent case–control studies have not demonstrated a significant association between contraceptive use and melanoma development [31,32].

The use of contraceptives, particularly exogenous hormones, has changed substantially over the years, with administration rates nearly doubling in 2015 compared to the 1970s. COs remain among the most widely used and generally safe methods of contraception [33,34]. However, findings from two randomized trials demonstrated a significant decline in the use of hormone therapy due to associated risks. These results suggested that, in some cases, the risks outweighed the benefits and could even increase the incidence of cardiovascular disease [35-37].

Coricovac *et al*. investigated the in vitro effects of EE, LG, and their combination on healthy skin cells and melanoma cells, both with and without UVB exposure. The results showed that at 1 µM, the hormones had no significant toxic effects on healthy cell lines but induced apoptosis in melanoma cell lines. At higher doses (10 µM), a cytotoxic effect was observed in all cell types, with a more pronounced impact on skin cancer cells. UVB irradiation was toxic to all cells, and the combination of UVB and hormonal stimulation resulted in synergistic cytotoxicity in A375 cells and increased the viability of healthy cells and of B164A5 murine melanoma cells. These results suggest a cell-type-dependent anti-melanoma effect, but further studies are needed to clarify the role of melanin in this context [38]. The data are consistent with those obtained by our research group.

Furthermore, research by Applebaum and colleagues has suggested a possible association between prolonged use of COs and an increased risk of developing squamous cell carcinoma. However, the study emphasizes the need for further investigation to confirm this association and to understand the biological mechanisms involved [39]. *In vitro* research has also examined the effects of combined contraceptives on breast cancer cell lines, highlighting varying influences on cell proliferation depending on hormone concentration and cell line type. A previous study conducted by our research team evaluated the cytotoxicity of EE and LG on breast cancer cells: MCF-7 and MDA-MB-231. The results exhibited that, after 24 hours of treatment with EE, LG, and the EE-LG combination, cell viability decreased with increasing doses of the compounds. However, extending the exposure period led to stimulation of cell proliferation. Notable differences were observed between the two cell lines: in MCF-7 cells, EE had a more pronounced stimulatory effect, while in the MDA-MB-231 line, LG induced cell proliferation and showed a reduced cytotoxic effect [40].

In another retrospective study, we investigated the risk factors involved in the progression of adenomyosis and the relationship between adenomyosis and breast cancer. Analyzing the clinical and demographic data of patients diagnosed with adenomyosis hospitalized over a certain period, it was observed that most cases occurred in women between 40 and 45 years of age. Factors such as multiparity, abortions, and a history of uterine surgery were associated with an increased risk of adenomyosis. Also, treatment with sex hormones, especially progestogens, was identified as a possible predisposing factor. To explore the link between adenomyosis and breast cancer, we evaluated the in vitro impact of LG on the breast cancer cells MDA-MB-231. The results pointed out that LG stimulated the proliferation of these cells in a dose- and time-dependent mode, suggesting a potential proliferative effect on breast carcinoma cells. These data emphasize the importance of careful evaluation of medical history and the use of hormonal treatments in patients with adenomyosis, given the possible link to breast cancer cell proliferation [41].

Another research by Simu *et al*. studied the impact of EE and LG on MDA-MB-231 breast carcinoma cells. The results showed that EE treatment reduced cell viability to 64.32% at 10 µM after 24 hours, caused morphological changes such as cell rounding, detachment from the plate, and loss of confluence, induced apoptotic aspects of the nuclei, and suppressed cell migration, suggesting a possible antitumor action. In contrast, LG induced proliferative activity, and the EE-LG combination had effects similar to EE, but less pronounced [42].

The impact of COs on melanoma has not been intensively studied; our research group has taken an important step in this direction. COs are effective pharmaceutical formulations, frequently prescribed as contraceptive methods, with beneficial effects on reproductive health.

Various current studies have investigated COs for actions other than those associated with cancer. Some studies have compared the endocrine activity of COs containing EE and those containing natural estrogen. Haverinen *et al*. reported that EE more strongly suppresses gonadotropin production than estradiol valerate [43], while Kangasniemi *et al*. demonstrated that EE-containing COs significantly alter adrenal steroidogenesis compared with estradiol-based preparations [44]. In addition, a 2022 study on vascular safety found that formulations containing LG–EE were associated with a lower risk of venous thromboembolism compared with those containing other progestogens [45]. We note that COs have been studied for several effects in both *in vitro* studies and clinical trials. Although some *in vitro* studies indicate that LG and EE could influence the development of murine melanoma, clinical evidence is insufficient and contradictory.

## CONCLUSION

Skin cancer is associated with high morbidity and mortality among women. Consequently, the link between this pathology and hormone exposure is of clinical interest, especially as the use of exogenous hormones has increased considerably in recent years. So far, there is little data to indicate that hormonal treatments and oral contraception influence the incidence of melanoma, this association being given by the doses used and the time of exposure. Following the analyses performed, we showed that the combination of COs did not negatively influence healthy skin cells (human or murine), and it had no significant impact on cell viability, LDH release, or morphological and nuclear integrity. On the other hand, a slight proliferation of murine B164A5 cells was observed with the increase in hormonal concentration, thus suggesting a possible relationship between melanoma development and the utilization of COs. Even so, further analysis is needed to understand the exact link between COs and melanoma.
